# Sleeping Habit and Other Life Styles in the Prime of Life and Risk for Ossification of the Posterior Longitudinal Ligament of the Spine (OPLL): a Case-control Study in Japan

**DOI:** 10.2188/jea.14.168

**Published:** 2005-03-18

**Authors:** Masakazu Washio, Gen Kobashi, Kazushi Okamoto, Satoshi Sasaki, Tetsuji Yokoyama, Yoshihiro Miyake, Naomasa Sakamoto, Kaori Ohta, Yutaka Inaba, Heizo Tanaka, Japan Collaborative Epidemiological Study Group for Evaluation of Ossification of the Posterio Longitudinal Ligament of the Spine (OPLL) Risk

**Affiliations:** 1 Department of Public Health, Sapporo Medical University School of Medicine.; 2 Department of Health for Senior Citizens, Hokkaido University Graduate School of Medicine.; 3 Department of Public Health, Aichi Prefectural College of Nursing and Health.; 4 Project of Scientific Evaluation of Dietary Reference Intakes, National Institute of Health and Nutrition.; 5 Department of Technology Assessment and Biostatistics, National Institute of Public Health.; 6 Department of Public Health, Fukuoka University School of Medicine.; 7 Department of Hygiene, Hyogo College of Medicine.; 8 Department of Epidemiology and Environmental Health, Juntendo University School of Medicine.; 9 National Institute of Health and Nutrition.

**Keywords:** Ossification of Posterior Longitudinal Ligament, Case-Control Studies, Risk Factors, Life Style, Sleep

## Abstract

BACKGROUND: Although the average age of onset of ossification of the posterior longitudinal ligamen of the spine (OPLL) is at around 50 years, the onset of the symptoms is insidious and the progression is very slow. The etiology of OPLL has not been elucidated in detail. Previous studies have suggested that a high-salt diet and low consumption of animal protein, glucose intolerance and high body mass are risk factors for OPLL. However, there is little information about the relationship between OPLL and life styles in the prime of life (between 30 and 50 years).

METHODS: To facilitate early prediction and prevention of OPLL, we analyzed life styles such as sleeping habit, physical exercise, smoking, alcohol drinking and hangover in subjects in the prime of life. Self-administered questionnaires were obtained from patients with OPLL and their sex- and age-matched controls. Sixty-nine patients diagnosed with OPLL within 3 years previously and 138 sex- and age-matched controls without backbone diseases, randomly selected from participants in a health checkup in a local town, were enrolled.

RESULT: Moderate amount of sleep (6-8 hours vs. 5 hours or shorter and 9 hours or longer; odds ratio [OR] = 0.18, 95% confidence interval [CI] = 0.06, 0.54) and a regular sleeping habit (i.e., going to bed and getting up at regular time) (OR=0.44, 95% CI=0.22, 0.90) were associated with a decreased risk o OPLL even after adjusting for other factors. On the other hand, moderate physical exercise (once a week or more v.s. less than once a week: OR=0.97, 95% CI=0.42, 2.26), smoking (OR=1.41, 95% CI=0.67, 2.97), drinking (OR=1.08, 95% CI=0.53, 2.20) and hangover (OR=1.12, 95% CI=0.43, 2.94) in the prime of life showed no correlation with risk of OPLL.

CONCLUSION: Good sleeping habits in the prime of life may decrease the risk of OPLL.

The Japanese Ministry of Health, Labour and Welfare has designated ossification of the posterior longitudinal ligament of the spine (OPLL) as an intractable disease because there is no established way to cure or prevent it.^1^ OPLL is manifested in the cervical vertebrae and causes a range of disease conditions varying from slight abnormality to quadriplegia.^2^^,^^3^ It has been called a Japanese disease because the prevalence is especially high in Japan.^4^ However, the relation of diffuse idiopathic skeletal hyperosteosis (DISH) to OPLL reported by Resnick et al.^5^ has recently attracted attention, and many cases of coexistence of the two diseases have been reported.^6^ Now, OPLL is recognized as an important disease occurring not only in Japan but also in Europe and in the United States.^7^ The prevalence is about 1.9% to 4.3% in general Japanese population.^2^^,^^4^ Males are twice^4^ or three times^8^ as likely to suffer from OPLL as females. Matsunaga et al.^4^ reported that the average age of onset of OPLL was 51 years for males and 49 years for females.

The etiology of OPLL has not yet been elucidated in detail, although it is thought to be a multifactorial disease in which complex environmental and genetic factors interact.^2^ Although many possible causative factors have been suggested, including genetic factors,^9^ sex,^10^ diabetes mellitus (DM),^10^^-^^12^ hypertension,^13^ high body mass index,^11^^,^^12^ injuries,^13^^,^^14^ hormonal imbalance,^15^ and dietary habits,^15^^,^^16^ the cause of the disease has not yet been clarified. Regarding dietary factors, previous epidemiologic studies carried out in Japan^15^ and Taiwan^16^ have suggested that both a high-salt diet and low consumption of animal protein are risk factors for OPLL. On the other hand, hospital-based studies in Japan^11^^,^^12^ revealed that both glucose intolerance and high body mass index were significantly associated with OPLL.

Among these risk factors, metabolic syndromes (i.e., insulin-resistance) including such as diabetes mellitus,^10^^-^^12^ glucose intolerance,^12^ and high body mass index^11^^,^^12^ may be important risk factors for OPLL from the point of view of prevention because they are also risk factors for lifestyle-related diseases such as cardiovascular diseases.^17^ Lifestyle modification such as physical exercise may reduce coronary risk^18^ by increasing insulin sensitivity. In contrast, sleeping few hours decreases the insulin-sensitivity^19^ and worsens the glucose intolerance.^20^ However, there is little information about the relationship between OPLL and life styles,^13^ even though lifestyles (e.g., physical exercise,^18^^,^^21^ alcohol drinking habits^18^^,^^22^ smoking habits,^18^ and sleeping habits^19^^,^^20^) are associated with the metabolic syndromes.

Although the average age of onset of OPLL is at around 50 years,^4^ the onset of the symptoms is insidious and the progression is very slow in most cases.^3^ OPLL may have a long period of latency. Thus, the present study was conducted to investigate the influence of sleeping habits and other lifestyle factors in the prime of life (between 30 and 50 years of age) on the development of OPLL.

## METHODS

Subjects were 69 patients who visited 9 collaborating hospitals in Hokkaido from 1998 through 2001, and who had been diagnosed as having OPLL in the previous 3 years. Diagnosis of OPLL was carried out by specialists on the basis of clinical symptoms and radiological examinations by using radiographs of the cervical and thoracic spines, tomography, computed tomography, and magnetic-resonance imaging according to the criteria of the Investigation Committee on the Ossification of the Spinal Ligaments, Japanese Ministry of Health and Welfare.^4^ All the patients were symptomatic and required medical consultation, and many of them underwent spinal surgery before or during the present study. Two controls matched to each case for sex and year of birth (within 3 years) without any backbone disorders were randomly recruited from participants in a health checkup in a town in Hokkaido. From 1998 through 2001, a self-administered questionnaire was obtained from the 69 OPLL patients and 138 controls. Written informed consent to cooperate in this study was obtained from all the subjects and controls. The contents of the questionnaire included (1) sleeping habits in the prime of life, (2) other lifestyles such as leisure time physical exercise, alcohol drinking and smoking in the prime of life and at the time of handling out the questionnarie, (3) height and body weight, and (4) past history of diseases such as diabetes mellitus. Many of the participants also agreed to donate blood samples which were stored until use for DNA extraction and genotyping of the candidate genes of OPLL. The details of the present study have been reported elsewhere.^11^

In univariate analysis, the differences were analyzed statistically by the chi-square test (degree of freedom = 1). Yates’ correction for continuity was used when the observed number was less than 5. A conditional logistic model was applied to evaluate the odds ratios (ORs) of lifestyle-related factors after adjusting for other risk factors (i.e., obesity, and diabetes mellitus). Because our previous report^11^ showed that diabetes mellitus and high body mass index (25 kg/m^2^ and over) were risk factors for OPLL, age, sex, and these two variables were used as other risk factors for OPLL to adjust the odds ratio in relation to the sleeping habit, leisure time physical exercise, smoking, alcohol drinking or hangover in multiple logistic regression analysis. Age was treated as a continuous variable, and indicator variables were used for other factors. All the statistical analyses were conducted by use of SAS^®^ package (SAS Institute Inc., Cary, NC).

The present study was approved by the institutional review boards of Hokkaido University School of Medicine and of each collaborating hospital.

## RESULTS

The mean (± standard deviation) ages of the 40 male cases and 80 male controls were 63.1 (± 9.6) years and 63.2 (± 9.5) years, respectively, and the mean ages of the 29 female cases and 58 female controls were 59.8 (± 10.1) years and 59.8 (± 10.1) years, respectively (not shown in the table).

Table 1 summarizes the ORs for OPLL and 95% confidence intervals in relation to lifestyles (i.e., sleeping habits, leisure time physical exercise, and smoking and drinking) in the prime of life. In univariate analysis, moderate (6-8 hours) sleeping hour (vs. short [5 hour or shorter] and excessive [9 hour or longer]) was associated with a decreased risk of OPLL (OR = 0.24, 95% confidence interval [CI] = 0.09, 0.59). So was a regular sleeping habit (i.e., going to bed and getting up at regular time) (vs. irregular sleeping habits; OR = 0.48, 95% CI = 0.27, 0.88). In contrast, insufficient sleep (i.e., the subjects felt that they had insufficient sleep) was associated with an increased risk of OPLL (vs. sufficient sleep; OR = 3.10, 95% CI = 1.31, 7.01). Even after adjusting for other factors such as age, sex, diabetes mellitus and high body mass index, moderate amount of sleep (OR = 0.18, 95% CI = 0.06, 0.54) and regular sleeping habit (OR=0.44, 95% CI=0.22, 0.90) were associated with a decreased risk of OPLL.

**Table 1.** Odds ratios (ORs) and 95% confidence intervals (CIs) for ossification of the posterior longitudinal ligament of the spine according to life style in the prime of life (between 30 and 50 years).

Factors		Crude OR (95% CI)	Adjusted OR (95% CI)
Sleeping hours			
Moderate (6-8 h/d) / Short (5h/d or less) and long ( 9h/d or more)		0.24 (0.09, 0.59)*^1^	0.18 (0.06, 0.54)*^7^
Had regular sleeping habits	Yes/ No	0.48 (0.27, 0.88)*^2^	0.44 (0.22, 0.90)*^8^
Had	Insufficient sleep/ Sufficient sleep	3.10 (1.31, 7.01)*^2^	1.85 (0.70, 4.91)*^8^
Moderate physical exercise			
	Once a week and more/ Less than once a week	0.86 (0.44, 1.70)*^3^	0.97 (0.42, 2.26)*^9^
Smoking habit	Current smokers/Never smokers, ex-smokers	1.37 (0.74, 2.54)*^4^	1.41 (0.67, 2.97)*^10^
Drinking habit	Once a week and more/ Less than once a week	1.03 (0.57, 1.88)*^5^	1.08 (0.53, 2.20)*^11^
Had a hangover	Once a week and more/ Less than once a week	6.86 (1.37, 32.24)*^6^	1.12 (0.43, 2.94)*^12^

Adjusted OR: adjusted for high body mass index (25.0 or greater) and diabetes mellitus.

* The number of cases and controls used for analysis.

*1:68 cases and 127 controls, *2: 68 cases and 133 controls, *3: 65 cases and 121 controls, *4:63 cases and 121 controls, *4: 67 cases and 127 controls, *5: 67 cases and 121 controls, *6: 57 cases and 104 controls, *7: 66 cases and 119 controls, *8: 66 cases and 122 controls, *9: 64 cases and 112 controls, *10: 60 cases and 114 controls, *11: 64 cases and 112 controls, *12: 57 cases and 98 controls

On the other hand, leisure time physical exercise in the prime of life did not show any significant relation to the risk of OPLL.

Smoking showed an adjusted OR greater than unity (current smokers vs. never smokers or ex-smokers; OR = 1.41, 95% CI = 0.67, 2.97) but failed to become a significant risk factor. There was no meaningful association between OPLL and alcohol drinking, while hangover was associated with an increased risk of OPLL (OR = 6.86, 95% CI = 1.37, 32.24). However, this positive association disappeared after adjusting for age, sex, diabetes mellitus, and high body mass index (OR = 1.12, 95% CI = 0.43, 2.94).

Table 2 shows the ORs and 95% CIs for OPLL according to sleeping hours in the prime of life. Short sleeping hours (5 hours or shorter) was associated with an increased risk of OPLL (vs. 6-8 hours; OR=6.74, 95% CI=2.08, 21.85). Even after adjusting for other factors such as age, sex, diabetes mellitus, and high body mass index, small amount of sleep (vs. 6-8 hours; OR=6.64, 95% CI=1.88, 23.49 ) was associated with an increased risk of OPLL. On the other hand, long sleeping hours (9 hours or longer) showed an increased OR (vs. 6-8 hours; OR=1.68, 95% CI=0.36, 7.79) and an adjusted OR greater than the unity (vs. 6-8 hours; OR=2.54, 95% CI=0.47, 13.65) but neither of them became a significant risk factor.

**Table 2.** Odds ratios (ORs) and 95% confidence intervals (CIs) for ossification of the posterior longitudinal ligament of the spine according to sleeping hours in the prime of life (between 30 and 50 years).

Factors	Cases	Controls	Crude OR (95% CI)	Adjusted OR (95%CI)
Sleeping hours				
Short (5 hours /day or shorter)	12	4	6.74 (2.08, 21.85)*^1^	6.64 (1.88, 23.49)*^1^
Moderate (6-8 hours /day)	53	119	1.00 (reference)	1.00 (reference)
Long ( 9 hours /day or longer)	3	4	1.68 (0.36, 7.79)*^2^	2.54 (0.47, 13.65)*^2^

Adjusted OR: adjusted for high body mass index (25.0 or greater) and diabetes mellitus.

* The number of cases and controls used for analysis.

*1:65 cases and 123 controls, *2: 56 cases and 123 controls

The participants also answered questions about their present drinking and smoking habits (Table 3). Current smokers did not differ between the two groups (40.0% vs. 30.4%, p=0.18: OR=1.56, 95% CI=0.81, 2.85). However, after adjusting for confounding factors such as age, sex, diabetes mellitus, and high body mass index, smoking showed an increased OR (OR=1.68, 95% CI=0.78, 3.63), but it did not reach significance. On the other hand, current drinkers were less common in the OPLL patients than in controls (30.3% vs. 64.2%, p<0.01: OR=0.29, 95%CI=0.15, 0.57). Even after adjusting for confounding factors such as age, sex, diabetes mellitus and high body mass index, current drinkers were less common among OPLL patients than among controls (OR=0.35, 95% CI=0.17, 0.75).

**Table 3.** Odds ratios (ORs) and 95% confidence intervals (CIs) for ossification of the posterior longitudinal ligament of the spine according to present status of smoking and drinking.

Factors		Crude OR (95% CI)	Adjusted OR (95%CI)
Smoking habit	Current smokers/Never smokers, ex-smokers	1.56 (0.81, 2.81)*^1^	1.68 (0.78, 3.63)*^3^
Drinking habit	Once a week and more/ Less than once a week	0.29 (0.15, 0.57)*^2^	0.35 (0.17, 0.75)*^4^

Adjusted OR: adjusted for high body mass index (25.0 or greater) and diabetes mellitus.

* The number of cases and controls used for analysis.

*1: 65 cases and 125 controls, *2: 66 cases and 120 controls, *3: 63 cases and 118 controls, *4: 64 cases and 113 controls

## DISCUSSION

The present study showed that bad sleeping habits in the prime of life were associated with an increased risk of OPLL though the average age of onset of OPLL is around 50 years.^4^ In the present study, moderate amount of sleep and regular sleeping habit in the prime of life were associated with a decreased risk of OPLL. These findings suggest that good sleeping habits in the prime of life may help prevent the development of OPLL.

In the present study, insufficient or excessive sleeping hour was more prevalent among OPLL patients than controls. Of the OPLL patients from the category who slept either too little or too much, 80% belonged to the former, whereas for the controls, this figure was 50%. Even after adjusting for other factors, small amount of sleep (5 hours or shorter) was associated with an increased risk of OPLL. On the other hand, sleeping long hours (9 hours or longer) showed an adjusted OR greater than the unity, but failed to become a significant risk factor. These findings suggest that sleeping few hours may be a risk factor for OPLL.

Although cigarette smoking and alcohol consumption are reported to have a negative relation to sleep duration,^23^ neither of these showed a significant relationship to the development of OPLL in the present study. In the present study, therefore, we did not adjust for smoking or drinking when evaluating the association between sleeping habits and the risk of OPLL.

The mechanism by which OPLL develops in persons with bad sleeping habits is still unclear. Chronic sleep deprivation predisposes to metabolic syndromes.^19^ Because diabetes mellitus,^10^^-^^12^ glucose intolerance,^12^ and high body mass index^11^^,^^12^ are reported as risk factors for OPLL, the insulin-resistance caused by bad sleeping habits may play a role in the development of OPLL. In the present study, however, even after statistical adjustment for diabetes mellitus and high body mass index, good sleeping habits such as sleeping an adequate but not excessive number of hours, and regular sleeping habit were associated with a decreased risk of OPLL.

Gonzalez-Ortiz et al.^24^ reported that 24-hour sleep deprivation decreased the insulin-sensitivity in healthy subjects. In addition, Spiegel et al.^20^ demonstrated that glucose tolerance decreased in the sleep-debt conditions. Furthermore, Vgontzas et al.^25^ pointed out that the indexes of sleep-disordered breathing were positively correlated with visceral fat but not body mass index among sleep-apnea patients. These findings and the results of the present study suggest that bad sleeping habits may cause insulin resistance, which may play an important role in the development of OPLL.

In the present study, current alcohol drinkers were less common in OPLL patients than controls even after adjusting for confounding factors such as diabetes mellitus and high body mass index. This result, however, should be interpreted with caution. First, the onset of the symptoms of OPLL is insidious and the progression is very slow in most cases.^3^ Second, in the present study, the proportion of current drinkers in the prime of life did not differ between OPLL patients and controls. In addition, the case control study by Nakamura et al.^13^ demonstrated that the proportion of drinkers who drank more than once a week did not differ between OPLL patients and controls. Furthermore, hangover in the prime of life was associated with an increased risk of OPLL before adjusting for confounding factors. We cannot deny the possibility that OPLL patients may have stopped drinking after the onset of their diseases. Further studies are needed to obtain a more accurate answer to this question.

Nakamura et al.^13^ reported that smoking showed an OR greater than unity (OR = 1.31, 95% CI = 0.77, 2.23) but did not reach significance. In the present study, smoking showed an increased OR (OR=1.68) but was not a significant risk factor, either. Our result was consistent with the result of the case control study by Nakamura et al.^13^ Further studies with large subject samples are required in order to evaluate the association between OPLL and smoking habit.

There are certain limitations to our study. First, our study is not free from information bias. OPLL patients may be more likely than controls to remember details of their lifestyles in the prime of life. In the present study, however, leisure time physical exercise, smoking and drinking in the prime of life were not associated with an increased risk of OPLL. In contrast, good sleeping habits such as sleeping a moderate number of hours and going to sleep and waking at regular hours in the prime of life were associated with a decreased risk of OPLL. These findings suggest that sleeping habits in the prime of life may be a more important factor than leisure time physical exercise, smoking or drinking in terms of the risk of OPLL in the prime of life.

Second, we obtained information about life style only in the prime of life and after the diagnosis of OPLL. Therefore, in the present study, we could not evaluate life style just before the diagnosis of OPLL. However, the average age of onset of OPLL is at around 50 years and the onset of the symptoms is insidious and progression is very slow. For these reasons, we suspected that life styles in the prime of life (between 30 and 50 years) may play the most prominent role in the development of OPLL.

Third, we did not obtain information about their activities of daily living (ADL). In the present study, however, the degree of disability in ADL may be similarly light among the patients because the progression of OPLL is very slow in most cases^3^ and all the patients were diagnosed as having the disease within 3 years.

Fourth, we matched controls to each case for sex and age only and not for proximity of residence. Although both OPLL patients and controls lived in Hokkaido prefecture, we cannot definitively deny that OPLL patients may have had different life styles from controls because they lived in towns other than the town where the health checkup was conducted.

Fifth, controls were recruited from participants in the town where the health checkup was conducted, and as such they may have been more health conscious than their OPLL counterparts. In order to confirm the result of the present study, we need to perform another case-control study in which the patients and controls are recruited in the same hospital.

Last, although OPLL is thought to be a multifactorial disease in which complex environmental and genetic factors interact,^2^^,^^9^ we did not evaluate the genetic factors in this paper.

On the other hand, our study has its strength as well. To the best of our knowledge, this case-control study is the first study to demonstrate an association between sleeping habits and OPLL.

In summary, the present study revealed that good sleeping habits in the prime of life such as sleeping a moderate number of hours and regular sleeping times were associated with a decreased risk for OPLL, while leisure time physical exercise, smoking and drinking in the prime of life did not show any meaningful relation to OPLL. The results of the present study suggest that bad sleeping habits in the prime of life may be a more important factor to increase the risk of OPLL than leisure time physical exercise, smoking or drinking in the prime of life. Since sleeping a moderate number of hours is negatively associated with mortality of all causes,^26^^-^^30^ good sleeping habits (i.e., going to bed and rising at approximately the same time everyday and sleeping for between 6 to 8 hours a day) should be recommended not only for the prevention of OPLL but also for longevity.

Part of this work was presented at the 13th annual meeting of Japan Epidemiological Association (January, 2003, Fukuoka, Japan).

## APPENDIX

Members of the Japan Collaborative Epidemiological Study Group for Evaluation of Ossification of the Posterior Longitudinal Ligament of the Spine (OPLL) Risk listed in alphabetical order for each affiliation: Hiroyuki Ohmori (Department of Public Health, Asahikawa Medical College), Masahiro Kokaji, Kiyoshi Kaneda (Center for Occupational Disorders and Injuries of the Spine, Bibai Rosai Hospital), Akira Hata (Department of Public Health, Chiba University Graduate School of Medicine), Motoyuki Shundo, Takeshi Masuda (Eniwa Hospital), Hiroyuki Anbo, Itaru Oda, Kyohichi Hasegawa (Hokkaido Orthopedics Memorial Hospital), Kazusa Shiozaki (Ito Orthopedics Hospital) , Hisashi Yoshimoto (Kitami Kobayashi Hospital), Takeshi Maeda (Department of Orthopedic Surgery, Kyushu University Graduate School of Medicine), Yoshimitsu Osawa, Hideo Watanabe (Nagoya Daiichi Red Cross Hospital), Makoto Kondo (NTT East Japan Sapporo Hospital) , Makoto Sugawara (Matsuda Orthopedics Hospital), Toshinori Nakao , Gou Takayama (Saga Insurance Hospital), Katsuhiro Aita, Takao Hotokebuchi (Department of Orthopedic Surgery, Saga Medical School), Toyoko Asami (Rehabilitation Center, Saga Medical School Hospital), and Shin-ichi Jokin (Shin-nittetsu Muroran Hospital).

## References

[1] Nakatani H. The advance and features of intractable diseases control as the health policy of the Japanese Ministry of Health and Welfare. In: Ohno Y, Tanaka H, Nakatani H, Kurokawa K, Saito H, eds. The latest information about intractable diseases, including epidemiology, clinical medicine and care for patients. Nanzando, Tokyo, 2000:3-27. (in Japanese)

[2] Ueyama K, Harada M. Ossification of the posterior longitudinal ligament (OPLL). In: Ohno Y, Tanaka H, Nakatani H, Kurokawa K, Saito H, eds. The latest information about intractable diseases, including epidemiology, clinical medicine and care for the patients. Nanzando, Tokyo, 2000:366-70. (in Japanese)

[3] Kawai S. Clinical manifestation of cervical ossification of the posterior longitudinal ligament. In: Yonenobu K, Sakou T, Ono K, eds. OPLL, ossification of the posterior longitudinal ligament. Springer-Verlag, Tokyo, 1997:81-84.

[4] Matsunaga S, Sakou T. Epidemiology of ossification of the posterior longitudinal ligament. In: Yonenobu K, Sakou T, Ono K, eds. OPLL, ossification of the posterior longitudinal ligament. Springer-Verlag, Tokyo, 1997:11-7.

[5] Resnick D, Shaul SR, Robins JM. Diffuse idiopathic skeletal hyperostosis (DISH): Forestier’s disease with extraspinal manifestations. Radiology 1975;115:513-24. 1129458 10.1148/15.3.513

[6] Resnick D, Niwayama G. Radiographic and pathologic features of spinal involvement in diffuse idiopathic skeletal hyperostosis (DISH). Radiology 1976;119:559-68. 935390 10.1148/119.3.559

[7] Koga H, Sakou T, Taketomi E, Hayashi K, Numasawa T, Harata S, . Genetic mapping of ossification of the posterior longitudinal ligament of the spine. Am J Hum Genet 1998;62:1460-7. 9585596 10.1086/301868 PMC1377147

[8] Ohtsuka K, Terayama K, Yanagihara M, Wada K, Kasuga K, Machida T, . An epidemiological survey on ossification of ligaments in the cervical and thoracic spine in individuals over 50 years of age. J Jpn Orthop Ass 1986;60:1087-98. 3102653

[9] Matsunaga S, Sakou T, Uehara H, Yamaguchi M, Koga H, Hayashi K. Genetic background of ossification of the posterior longitudinal ligament. In: Yonenobu K, Sakou T, Ono K, eds. OPLL, ossification of the posterior longitudinal ligament. Springer-Verlag, Tokyo, 1997:19-25.

[10] Tsuyama N. Ossification of the posterior longitudinal ligament of the spine. Clin Orthop 1984;184:71-84. 6423334

[11] Kobashi G, Washio M, Okamoto K, Sasaki S, Yokoyama T, Miyake Y, . High body mass index after age 20 and diabetes mellitus are independent risk factors for ossification of the posterior longitudinal ligament of the spine in Japanese subjects: A case-control study in multiple hospitals. Spine 2004;29:1006-10. 15105673 10.1097/00007632-200405010-00011

[12] Shingyouchi Y, Nagahama A, Niida M. Ligamentous ossification of the cervical spine in the late middle-aged Japanese men. Its relation to body mass index and glucose metabolism. Spine 1996;21:2474-8. 8923634 10.1097/00007632-199611010-00013

[13] Nakamura Y, Ohshiro H, Nose T, Hossaka K, Yamamoto M, Omura T, . A case-control study of ossification of the posterior longitudinal ligament of spine in Japan. J Epidemiol 1995;5:29-33.

[14] Katoh S, Ikata T, Hirai N, Okada Y, Nakauchi K. Influence of minor trauma to the neck on the neurological outcome in patients with ossification of the posterior longitudinal ligament (OPLL) of the cervical spine. Paraplegia 1995;33:330-3. 7644259 10.1038/sc.1995.74

[15] Musha Y. Etiological study of spinal ligament ossification with special reference to dietary habits and serum sex hormones. J Jpn Orthop Assoc 1990;64:1059-71. (in Japanese) 2125635

[16] Wang PN, Chen SS, Liu HC, Fuh JL, Kuo BI, Wang SJ. Ossification of the posterior longitudinal ligament of the spine. A case-control risk factor study. Spine 1999;24:142-4. 9926384 10.1097/00007632-199901150-00010

[17] Farmer JA, Gotto AM. Dyslipidemia and other risk factors for coronary artery disease. In: Braunwald E, eds. Heart Disease, a Textbook of Cardiovascular Medicine, 5th ed. WB Saunders Companay. Philadelphia, 1997:1126-60.

[18] Kaplan NM. Treatment of hypertension: lifestyle modification. In: Kaplan NM, eds. Kaplan’s Clinical Hypertension, 8th ed. Lippincott Williams and Wilkins. Philadelphia, 2002:206-36.

[19] Scheen AJ. Clinical study of the month. Does chronic sleep deprivation predispose to metabolic syndrome? Rev Med Liege 1999;54:898-900. (in French) 10667051

[20] Spiegel K, Leproult R, Van Cauter E. Impact of sleep debt on metabolic and endcrine function. Lancet 1999;354:1435-9. 10543671 10.1016/S0140-6736(99)01376-8

[21] Mascioli EA, Bistrian BR. Treatment of obesity. In: Kahn CR, Weir GC, eds. Joslin’s Diabetes Mellitus, 13th ed. Williams and Wilkins. Baltimore, 1994:363-71.

[22] Quickel KE Jr. Economic and social cost of diabetes. In: Kahn CR, Weir GC, eds. Joslin’s Diabetes Mellitus, 13th ed. Williams and Wilkins. Baltimore, 1994:586-604.

[23] Palmer CD, Harrison GA, Hirons RW. Association between smoking and drinking and sleep duration. Ann Hum Biol 1980;7:103-7. 7425536 10.1080/03014468000004111

[24] Gonzales-Ortiz M, Martinez-Abundis E, Balcazar-Munoz BR, Pascoe-Gonzalez S. Effect of sleep deprivation on insulin sensitivity and cortisol concentration in healthy subjects. Diabetes Nutr Metab Clin Exp 2000;13:80-3. 10898125

[25] Vgontzas AN, Papanicolaou DA, Bixler EO, Hopper K, Lotsikas A, Lin HM, . Sleep apnea and daytime sleepiness and fatigue: relation to visceral obesity, insulin resistance, and hypercytokinemia. J Clin Endcrinol Metab 2000;85:1151-8. 10.1210/jcem.85.3.6484 10720054

[26] Breslow L. Risk factor intervention for health maintenance. Science 1978;26:908-12. 10.1126/science.644333 644333

[27] Kripke DF, Garfinkel L, Wingard DL, Klauber MR, Marler MR. Mortality associated with sleep duration and insomnia. Arch Gen Psychiatry 2002;59:131-6. 11825133 10.1001/archpsyc.59.2.131

[28] Kojima M, Wakai K, Kawamura T, Tamakoshi A, Aoki R, Lin Y, . Sleep patterns and total mortality: a 12-year follow-up study in Japan. J Epidemiol 2000;10:87-93. 10778032 10.2188/jea.10.87

[29] Tamakoshi A, Ohno Y. Self-reported sleep duration as a predictor of all-cause mortality: results from the JACC study, Japan. Sleep 2004;27:51-4. 14998237

[30] Amagi Y, Ishikawa S, Gotoh T, Doi Y, Kayaba K, Nakamura Y, . Sleep duration and mortality in Japan: the Jichi Medical Chort Study. J Epidemiol 2004;14:124-8. 15369129 10.2188/jea.14.124 PMC8702369

